# Breast cancer burden in the United States (1990–2021) with a 15-year forecast: a comprehensive analysis based on the global burden of disease 2021

**DOI:** 10.3389/fonc.2025.1650110

**Published:** 2025-11-13

**Authors:** Zhijian Huang, Lingxiao Zhang, Peizhang Liu, Hongxiang Lin, Zeyi Peng, Peixin Zheng, Xinhao Sun, Lisheng Lin

**Affiliations:** 1 Department of Breast Surgical Oncology, Clinical Oncology School of Fujian Medical University, Fujian Cancer Hospital, Fuzhou, China; 2 Cancer Research Institute, Beth Israel Deaconess Medical Center, Harvard Medical School, Boston, MA, United States; 3 Division of Rheumatology, Allergy, and Immunology, Department of Medicine, Massachusetts General Hospital, Boston, MA, United States; 4 College of Science, Northeastern University, Boston, MA, United States; 5 Department of Breast Surgery, The Affiliated Hospital of Putian University, Putian, Fujian, China

**Keywords:** breast cancer, global burden of disease, epidemiology, socio-demographic index, U.S.

## Abstract

**Objective:**

Breast cancer (BC) is one of the most common cancers globally, placing a significant social burden. This study estimates the BC burden in the U.S. from 1990 to 2021 and projects future trends for the next 15 years.

**Methods:**

Using data from the Global Burden of Disease (GBD) 2021 study, we analyzed four measures: prevalence, incidence, death, and disability-adjusted life years (DALYs), stratified by sex, age, U.S. states, and socio-demographic index (SDI).

**Results:**

BC burden in the U.S. has decreased, with reductions in age-standardized rates of prevalence, incidence, mortality, and DALYs for both sexes. The overall age-standardized prevalence rate dropped from 695.0 (653.5–741.5)/100,000 in 1990 to 556.0 (525.2–584.7)/100,000 in 2021. The ASIR declined from 68.3 (65.1–70.3)/100,000 to 51.7 (48.4–54.1)/100,000. Death rates fell from 15.9 (14.9–16.5)/100,000 to 9.4 (8.5–9.9)/100,000, while DALYs decreased from 485.1 (462.9–507.0)/100,000 to 277.4 (260.1–294.8)/100,000 over the same period. Burden varies by state and SDI: in 2021, low-SDI states, Kentucky and Louisiana had the highest prevalence and incidence, while Louisiana and Mississippi had the highest mortality. Projections suggest a continued downward trend through 2036.

**Conclusions:**

BC burden in the U.S. decreased overall, but disparities persist across sex, age groups, and states with varying SDI levels. Addressing risk factors and improving healthcare access are essential to further reduce BC burden.

## 1 Introduction

Breast cancer (BC) typically develops in the breast lobules, ducts, or connective tissue, often leading to metastasis if left undetected and untreated, which can be fatal (1, 2). Worldwide, it has been identified as the second most commonly diagnosed cancer and the fourth leading cause of cancer mortality according to the GLOBOCAN 2022 data (3). In the U.S., BC has also continuously been a burdensome chronic disease. In 2023, an estimated 297,790 new BC cases were diagnosed among women in the U.S., representing approximately one-third of all female cancers. Additionally, 43,170 BC-related deaths were projected, accounting for 15% of all cancer deaths, according to the 2023 U.S. cancer statistics. Although the incidence rates of BC in women have been gradually rising since the mid-2000s, mortality rates have declined by 43% after a peak reached in 1989. This reduction in mortality burden could be attributed to earlier detection through mammography screenings, improved public awareness, and advances in treatments (4–6).

BC incidence and mortality varies by different demographics (6). Even though the incidence of BC is much higher in women than in men (7, 8), with 1 in 8 for women and 1 in 1000 for men during lifetime, the trend is still increasing in males (9). Additionally, the risk of developing BC increases with age (10). BC has multiple genetic and environmental risk factors (11–13), and the U.S. variation in population demographics and regional characteristics in a country can lead to health disparities in BC burden. Specifically, the differences in economic development across U.S. states highlight the need for improved healthcare resource allocation and more effective health policies to address health inequalities nationwide.

Therefore, it becomes important to examine the trends of BC incidence and mortality in the U.S. to better monitor and understand the BC burden, and their changes by key demographic features such as age, sex, and socio-demographic index (SDI) at national level and state level. Moreover, our findings resonate with those of the Global Burden of Disease (GBD) 2019 Breast Cancer Collaborators and the Women’s Cancer Systematic Analysis, collectively underscoring the central role of the SDI in shaping disease burden. This convergence provides a robust global context for our state-level dissection of disparities across the United States. The GBD is a systematic scientific enterprise that quantifies health loss worldwide through standardized methods. Delivering comparable estimates for 371 diseases and injuries and 88 risk factors across 204 countries and territories, the GBD generates metrics such as disability-adjusted life years (DALYs) that integrate premature mortality and years lived with disability into a single composite measure (14, 15). While previous study only used data up to 2019 or lacked a thorough assessment the BC burden focusing on the U.S (16–18). In this study, we aimed to provide the most recent estimates of the BC burden in the U.S. by incorporating the latest iteration of the GBD 2021 study data. Thus, the study can contribute to the enhancement of preventive efforts, reducing the U.S. and global disease burden, and informing public health policy and practice.

## 2 Method

### 2.1 Data source

The GBD study offers detailed and standardized health estimates on the impact of diseases and injuries across the world. The most recent iteration, GBD 2021, provides a robust analysis of 371 diseases and injuries, as well as 88 risk factors, covering 204 countries and territories from 1990 to 2021 (19). BC data, along with insights on other health burdens, can be accessed through the Global Health Data Exchange (GHDx) online query tool (http://ghdx.healthdata.org/gbd-results-tool). This includes information on prevalence rate, age-standardized incidence rate (ASIR), age-standardized death rate (ASDR), years of life lost (YLLs), years lived with disability (YLDs), and DALYs from 1990 to 2021 in the United States, at both national and state levels.

### 2.2 Estimation of BC burden and prediction

The GBD 2021 study primarily employed the DisMod-MR 2.1 model for estimation. DisMod-MR 2.1 is a Bayesian meta-regression tool capable of integrating epidemiological data from diverse sources and of varying quality. It incorporates hierarchical models to account for correlations across different geographical regions and population subgroups, thereby generating internally consistent and cross-nationally comparable estimates of disease rates. For mortality data, GBD 2021 utilized the Cause of Death Ensemble model (CODEm) and cause-specific modeling strategies, which synthesize information from death registration systems, verbal autopsy data, and other sources. To smooth data over spatial and temporal dimensions and impute missing values, the study extensively applied Spatio-temporal Gaussian Process Regression (ST-GPR). This technique captures continuous geographical and temporal variation patterns and autocorrelations in disease burden, thereby enhancing the robustness and reliability of the estimates. In assessing the contribution of risk factors to disease burden, GBD 2021 adopted the Comparative Risk Assessment (CRA) framework to calculate the proportion of disease burden attributable to specific risk factors (20, 21). Among the metrics used to estimate the burden of BC, DALYs encompass both YLLs and YLDs from prevalent cases within a population (22–24). Consequently, YLLs and YLDs were not discussed specifically in this study, and we focused on DALYs.

These BC burden measurements are analyzed by 16 age groups (from 15 to 94 years old with 5-year intervals), by gender, by states, and by SDI. SDI serves as a crucial indicator for assessing the level of development of countries and regions. This index includes factors such as per capita gross national income, total fertility rates for populations under 25 years of age, and the average years of education for individuals aged 15 and older (25). In this study, we also showed the relationship between BC burden estimates and the SDI of various countries and regions worldwide. This comparison offers valuable insights into the broader influence of SDI on global BC burden, allowing us to evaluate how the U.S. aligns with worldwide trends. It also helps assess the U.S.’s position relative to other nations and how its preventive efforts have impacted that standing.

Additionally, we employed the Auto-Regressive Integrated Moving Average (ARIMA) model to predict age-standardized rates from 2022 to 2036, leveraging its ability to capture trends, autocorrelations, and seasonality in the data. Trained on the observed data, the model provided 15-year projections, including point estimates and 95% confidence intervals, calculated from the associated standard errors. Forecasts were generated for each year following the last recorded year, with both the point estimates and confidence intervals reported to reflect the uncertainty of the predictions.

### 2.3 Statistical analysis

This study reports crude all-age numbers for the burden estimation measurements and age-standardized rates, which were calculated using the direct method of standardization based on the GBD world population standard, reported/100,000 population, along with the 95% uncertainty intervals (UI) of numbers and rates. Polynomial regression models were employed to measure the temporal trends of all-age numbers and age-standardized rates of certain burden metrics, including prevalence, incidence, death, and DALYs. A two-tailed p-value of 0.05 was considered statistically significant. All analyses were conducted using R software (version 4.4.1).

## 3 Results

### 3.1 Breast cancer prevalence and incidence

**Figure 1:**
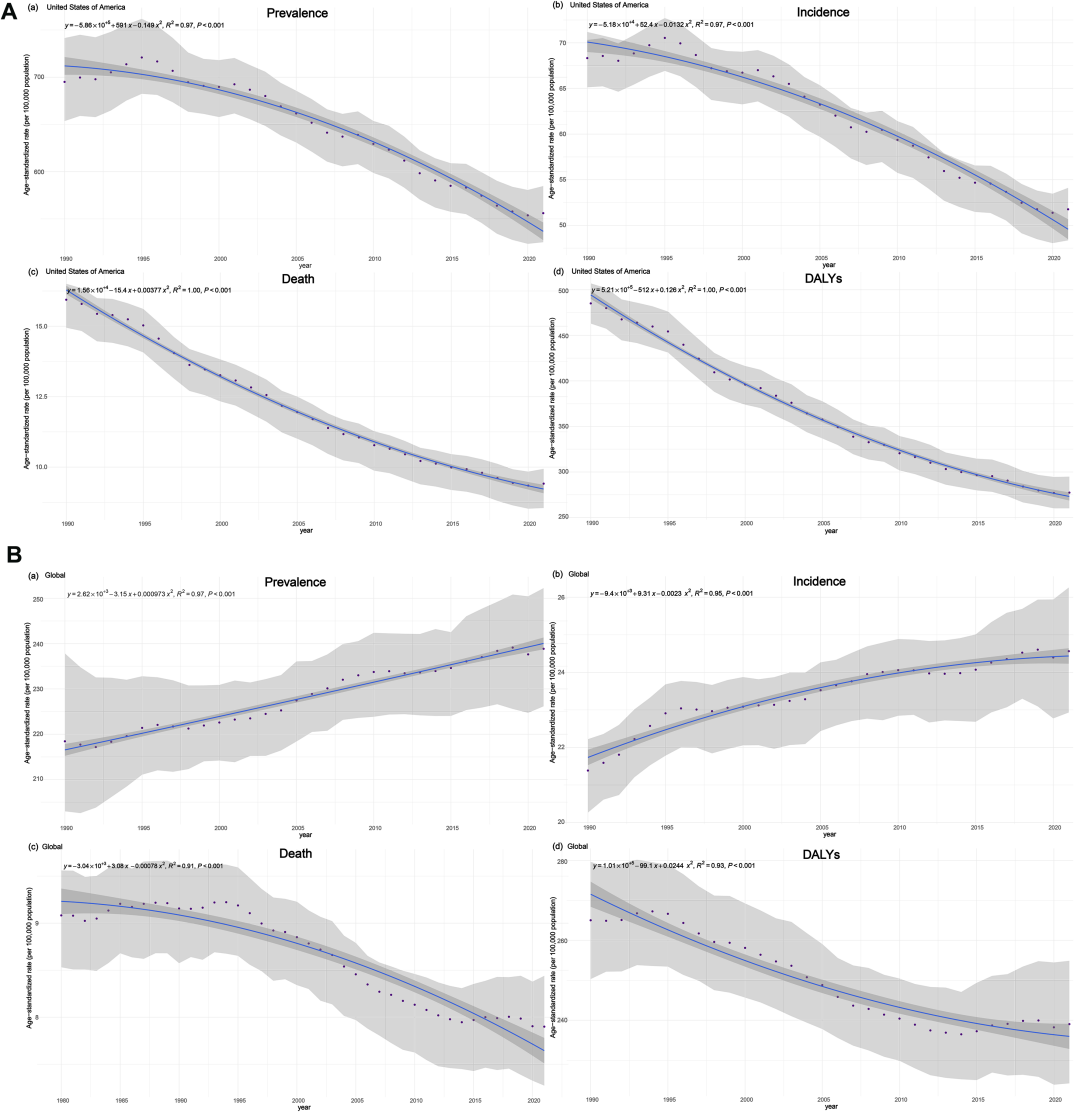
Time trends of BC age-standardized incidence, prevalence, deaths and DALYs, rates (per 100,000 population) and 95% UIs, in the U.S. **(A)** and globally **(B)**, from 1990 to 2021 for both sexes.

Overall, the number of new BC developed in the U.S. for both males and females were 204,920 (194,242 - 211,265) in 1990, rising to 272,387 (251,345 - 285,258) in 2021, while the ASIR dropped from 68.3 (65.1 - 70.3)/100,000 to 51.8 (48.4 - 54.1)/100,000 in this period. Additionally, the age-standardized prevalence rate decreased from 695.0 (653.5 - 741.5)/100,000 in 1990 to 556.0 (525.2 - 584.7)/100,000 in 2021, even though absolute count of cumulative cases expanded from 2,111,669 (1,973,086 - 2,266,808) in 1990 to 2,999,127 (2,813,937 - 3,167,566) in 2021 (**Table 1**). To summarize, numbers of existing and newly diagnosed BC cases both showed increasing trends from 1990 to 2021 for both sexes, while the age-standardized rates for prevalence and incidence, unlike the increasing global trends (**Figure 1B**), both decreased within this time (**Figure 1A**).

**Table 1:** BC incidence, prevalence, deaths, DALYs, YLLs, and YLDs in the US in 1990 and 2021, for both sexes, females, and males.

Measure	Metric	1990			2021		
		Both	Female	Male	Both	Female	Male
Prevalence	Number	2111669 (1973086 - 2266808)	2091717 (1954147 - 2245625)	19953 (18675 -	2999127 (2813937 - 3167567)	2965312 (2781117 - 31329345)	33816 (32030 - 35296)
	Rate	695.0 (653.5 - 741.5)	1250.5 (1181.2 - 1329.8)	14.9 (14.0 - 16.0)	556.0 (525.2 - 584.7)	1037.5 (982.0 - 1091.0)	13.0 (12.3 - 13.6)
Incidence	Number	204920 (1942412 - 211265)	202852 (192228 - 209178)	2068 (1987 - 2137)	272388 (251345 - 285258)	269012 (248072 - 281855)	3375 (3202 - 3536)
	Rate	68.3 (65.1 - 70.3)	124.3 (119.0 - 127.7)	1.5 (1.5 - 1.6)	51.7 (48.4 - 54.1)	97.0 (91.0 - 101.3)	1.3 (1.2 - 1.4)
Deaths	Number	49188 (45894 - 50999)	48806 (45525 - 50614)	381 (366 - 394)	53473 (47933 - 56791)	52869 (47359 - 56161)	603 (565 - 633)
	Rate	15.9 (14.9 - 16.5)	28.2 (26.6 - 29.1)	0.3 (0.3 - 0.3)	9.4 (8.5 - 9.9)	17.2 (15.7 - 18.1)	0.2 (0.2 - 0.2)
DALYs	Number	1423204 (1355188 - 1488967)	1412061 (1344370 - 1477199)	11142 (10574 -	1423646 (1324856 - 1521530)	1407343 (1309216 - 1504084)	16303 (15212 - 17524)
	Rate	485.1 (462.9-507.0)	894.0 (855.5 - 933.1)	8.4 (7.9- 8.9)	277.4 (260.1 - 294.8)	522.4 (490.8- 554.9)	6.4 (5.9 - 6.8)
YLDs	Number	151600 (108685 - 201890)	149862 (107466 - 199629)	1738 (1259 - 2299)	205394 (146573 - 274819)	202542 (144486 - 271033)	2853 (2059 - 3791)
	Rate	50.2 (35.9 - 67.2)	90.8 (64.8 - 121.5)	1.3 (0.9 - 1.7)	38.8 (27.6 - 52.0)	72.5 (51.6 - 97.1)	1.1 (0.8 - 1.5)
YLLs	Number	1271604 (1218459 - 1305095)	1262199 (1209351 - 1295693)	9405 (9100 - 9672)	1218252 (1140966 - 1274615)	1204801 (1128125 - 1260902)	13451 (12838 - 14020)
	Rate	434.9(418.4 -445.8)	803.2 (775.2 - 822.5)	7.1(6.8 - 7.3)	238.6 (225.2 - 248.6)	449.9(425.9 - 468.2)	5.3 (5.0 - 5.5)

Data in parentheses are 95% confidence intervals.

*Age-standardized rate per 100,000.

** All ages.

When analyzing by sex, females represented the majority of new BC cases. In 1990, women were diagnosed with 202,852 (192,228 - 209,178) new cases, accounting for approximately 99% of all new cases that year. By 2021, this number rose to 269,012 (248,072 - 281,855), still representing around 99% of all new BC cases for both sexes. The age-standardized prevalence rate for females was 1250.5 (1181.2 - 1329.8)/100,000 in 1990 and 1037.5 (982.0 - 1091.0)/100,000 in 2021. The ASIR for females was 124.3 (119.0 - 127.7)/100,000 in 1990 and 97.0 (91.0 - 101.3)/100,000 in 2021. For males, the rates were significantly lower, with the prevalence rate decreasing from 14.9 (14.0 - 16.0)/100,000 in 1990 to 13.0 (12.3 - 13.6)/100,000 in 2021, and the ASIR decreasing from 1.5 (1.5 - 1.6)/100,000 in 1990 to 1.3 (1.2 - 1.4)/100,000 in 2021 (**Table 1**). Both prevalence and incidence rates for females and males showed a downward trend, consistent with the overall tendency for both sexes.

**Figure 2:**
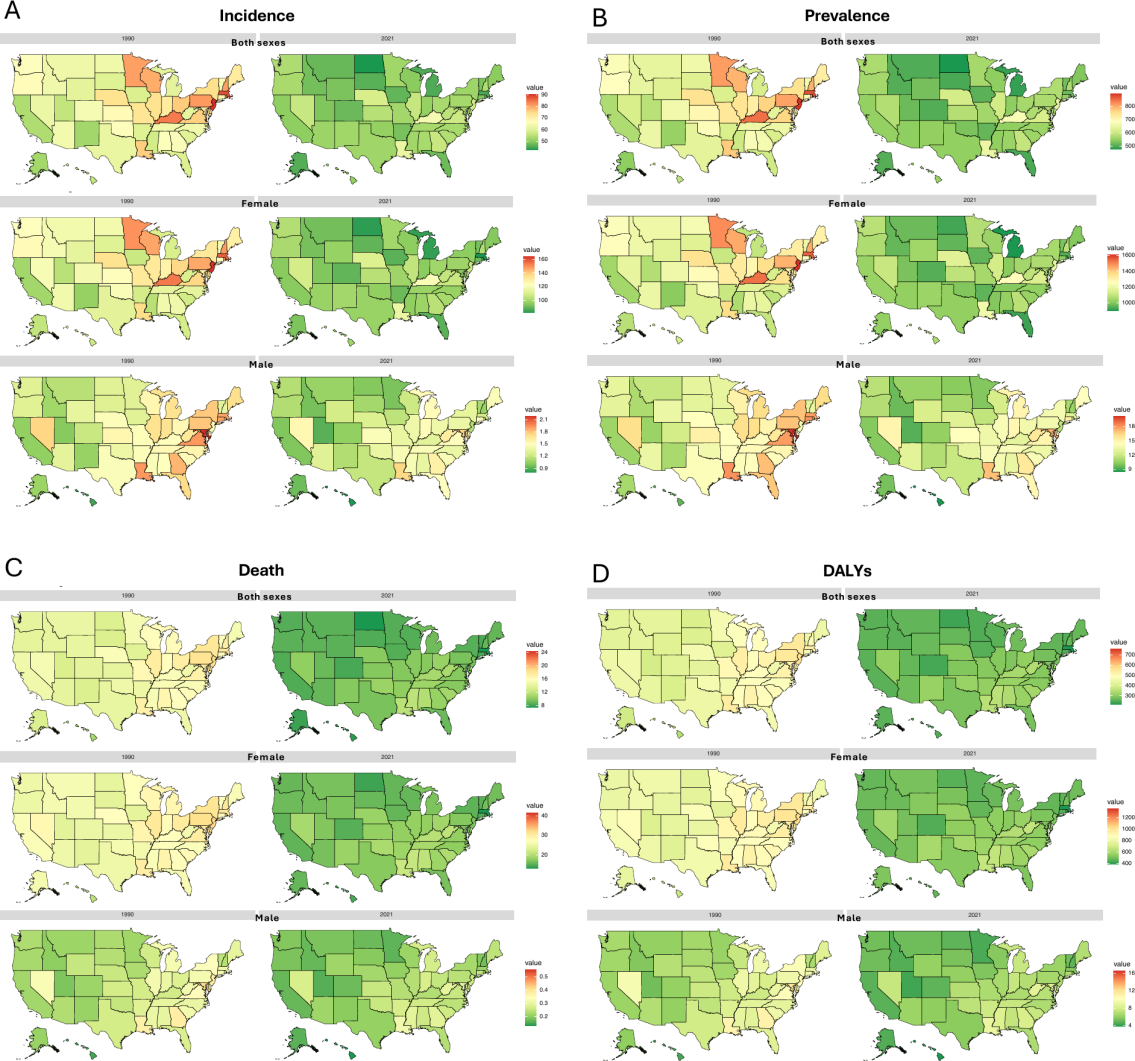
BC age-standardized incidence **(A)**, prevalence **(B)**, death **(C)**, and DALYs **(D)** rates (per 100,000) at the US-state level in 1990 and 2021, by sex groups.

Across the U.S., similar trends were observed at the subnational level, with all states showing a decrease in BC ASIR and age-standardized prevalence rate for both males and females if comparing 1990 and 2021, as demonstrated in the US maps in **Figures 2A, B**. In 1990, the highest and lowest estimated ASIR for both sexes among all provinces were 90.2 (83.2 - 97.0)/100,000 in New Jersey and 52.3 (47.1 - 57.0)/100,000 in New Mexico, respectively, while in 2021, the highest and lowest rates were 63.9 (52.3 - 78.9)/100,000 in Kentucky and 42.3 (35.7 - 50.5)/100,000 in North Dakota, respectively. Focusing on females, New Mexico state had the lowest ASIR, 96.9 (87.1 - 105.5)/100,000 in 1990, while New Jersey state had the highest rate of 163.4 (150.9 - 175.8)/100,000. Moving to 2021, the minimum rate was observed in Massachusetts 82.3 (62.4 - 102.3)/100,000, and the maximum rate was 120.4 (98.1 - 149.5)/100,000 in Kentucky (**Supplementary Table 1**).

In 1990, the highest age-standardized prevalence rate was recorded in New Jersey at 891.5 (821.4 - 963.8)/100,000, while Alaska had the lowest at 543.7 (490.2 - 596.7)/100,000. Among females, rates ranged from the lowest rate of 1012.2 (919.8 - 1110.3)/100,000 in New Mexico to the highest rate of 1600.9 (1475.2 - 1720.7)/100,000 in New Jersey. For males, the lowest rate was 9.5 (8.2 - 10.8)/100,000 in Hawaii, and the highest was 20.2 (17.3 - 23.3)/100,000 in Maryland. By 2021, the lowest rate overall was in North Dakota at 475.9 (420.3 - 546.3)/100,000. For females, the lowest rate was in Michigan at 902.3 (747.4 - 1064.2)/100,000, and for males, it was in Hawaii at 8.4 (6.6 - 10.2)/100,000. The highest rates in 2021 were observed in Kentucky overall at 671.0 (576.5 - 788.9)/100,000, with the highest rate for females also in Kentucky at 1258.7 (1076.0 - 1487.9)/100,000 and for males still in Maryland at 17.4 (14.0 - 21.6)/100,000 (**Supplementary Table 1**). Data on all-age numbers of all burden measures in 1990 and 2021 of all U.S. states were listed in **Supplementary Table 2**. We also created novel animations to vividly illustrate the changes in incidence and prevalence rates from 1990 to 2021 of all U.S. states, separately by sex groups (both sexes, females, and males), which can be found in **Supplementary Figures 1, 2**.

### 3.2 Breast cancer mortality

At national level, the age-standardized DALYs rate for females was 894.0 (855.5 - 933.1)/100,000 in 2021, down from 522.4 (490.8 - 554.9)/100,000 in 1990, reflecting a 71.1% decrease. A similar trend was observed for males, although the decline was smaller, with the age-standardized DALYs rate in 2021 at 6.4 (5.9 - 6.8)/100,000 compared to 8.4 (7.9 - 8.9)/100,000 in 1990, indicating a 31.3% decrease. Nationally, the overall DALYs rate decreased from 485.1 (462.9 - 507.0)/100,000 in 1990 to 277.4 (260.1 - 294.8)/100,000 in 2021 (**Table 1**). This downward trend in both age-standardized DALYs rates and ASDR for both sexes over the 31 years is evident, though the numbers of the two measures did not consistently decline throughout the period (**Figure 1B**), with recent increases observed following years of decreasing. Nonetheless, the BC mortality trend in the U.S. generally aligns with the global pattern (**Figure 1A**), where age-standardized death rates and DALYs decreased from 1990 to 2021. In 1990, there were 49,188 (45,894 - 50,999) deaths attributed to BC nationwide, while in 2021, this number increased to 53,473 (47,933 - 56,791). Among females, BC caused 48,806 (45,525 - 50,614) deaths in 2021, a decrease from the 52,869 (47,359 - 56,161) deaths recorded in 1990. Female death cases accounted for approximately 99% of all deaths for both sexes in both years and only about 1% of BC-induced deaths were from men. However, the number of BC-related deaths among males increased over the same period. In 2021, there were 603 (565 - 633) deaths, reflecting a 58.2% ([603-381]/381) increase from the 381 (366 - 394) deaths in 1990. Despite the rise in male mortality, the overall burden of BC-related deaths remains significantly higher in women than in men. In 1990, the ASDR for both sexes were 15.9 (14.9 - 16.5)/100,000, with females at 28.2 (26.6 - 29.1)/100,000 and males at 0.3 (0.3 - 0.3)/100,000. Notably, by 2021, the ASDR had decreased for both sexes, with rates of 9.4 (8.5 - 9.9)/100,000 overall, 17.2 (15.7 - 18.1)/100,000 for females, and 0.2 (0.2 - 0.2)/100,000 for males (**Table 1**).

The patterns and changes in ASDR and age-standardized DALYs rates across all US states are illustrated in **Figures 2C, D** for both sexes, females, and males, comparing 1990 and 2021. In 1990, the highest ASDR across states was 24.2 (22.3 - 25.9)/100,000 in the District of Columbia, while the lowest was 10.9 (9.9 - 11.8)/100,000 in Hawaii. By 2021, this pattern had shifted, with the highest ASDR recorded in Mississippi at 11.9 (9.6 - 14.6)/100,000 and the lowest in Massachusetts at 7.4 (5.7 - 9.1)/100,000. For females, Hawaii had the lowest ASDR in 1990 at 20.7 (18.9 - 22.4)/100,000, while the District of Columbia had the highest rate at 41.4 (38.5 - 44.4)/100,000, which was somehow consistent with the overall national pattern in that year. By 2021, the lowest ASDR was also found in Massachusetts at 13.2 (10.1 - 16.2)/100,000, while the highest was in Louisiana at 21.5 (17.4 - 26.0)/100,000 (**Supplementary Table 1**).

In terms of DALYs, in 1990, the District of Columbia recorded the highest DALYs rate at 758.9 (707.8 - 818.7)/100,000, while Hawaii had the lowest at 347.9 (317.8 - 378.6)/100,000. Interestingly, the District of Columbia also had the highest rates for both women and men, at 1356.5 (1262.8 - 1465.7)/100,000 and 16.5 (14.7 - 18.6)/100,000, respectively. Similarly, Hawaii again reported the lowest rates for both genders, with 670.8 (613.4 - 729.9)/100,000 for women and 4.4 (3.9 - 5.0)/100,000 for men. By 2021, the trend shifted with Massachusetts reporting the lowest DALYs rate at 210.1 (161.8 - 256.0)/100,000, and Mississippi the highest at 353.9 (283.3 - 434.4)/100,000 for both sexes. This year, the gender-specific patterns diverged from the national trend: Louisiana had the highest age-standardized DALYs rate for women at 662.3 (535.7 - 809.8)/100,000, while the District of Columbia had the highest for men at 9.5 (7.4 - 12.2)/100,000. For 2021, the lowest DALYs rate for females was observed in Massachusetts at 387.4 (296.1 - 482.7)/100,000, and for males, it was in Hawaii at 3.8 (2.9 - 4.9)/100,000 (**Supplementary Table 1**). Data on all-age numbers of all burden measures in 1990 and 2021 of all U.S. states were listed in **Supplementary Table 2**. The novel animations demonstrating the changes in death and DALYs rates across all U.S. states by sex (both sexes, females, and males) from 1990 to 2021 which can be found in **Supplementary Figures 3, 4**.

### 3.3 Breast cancer burden by age

**Figure 3:**
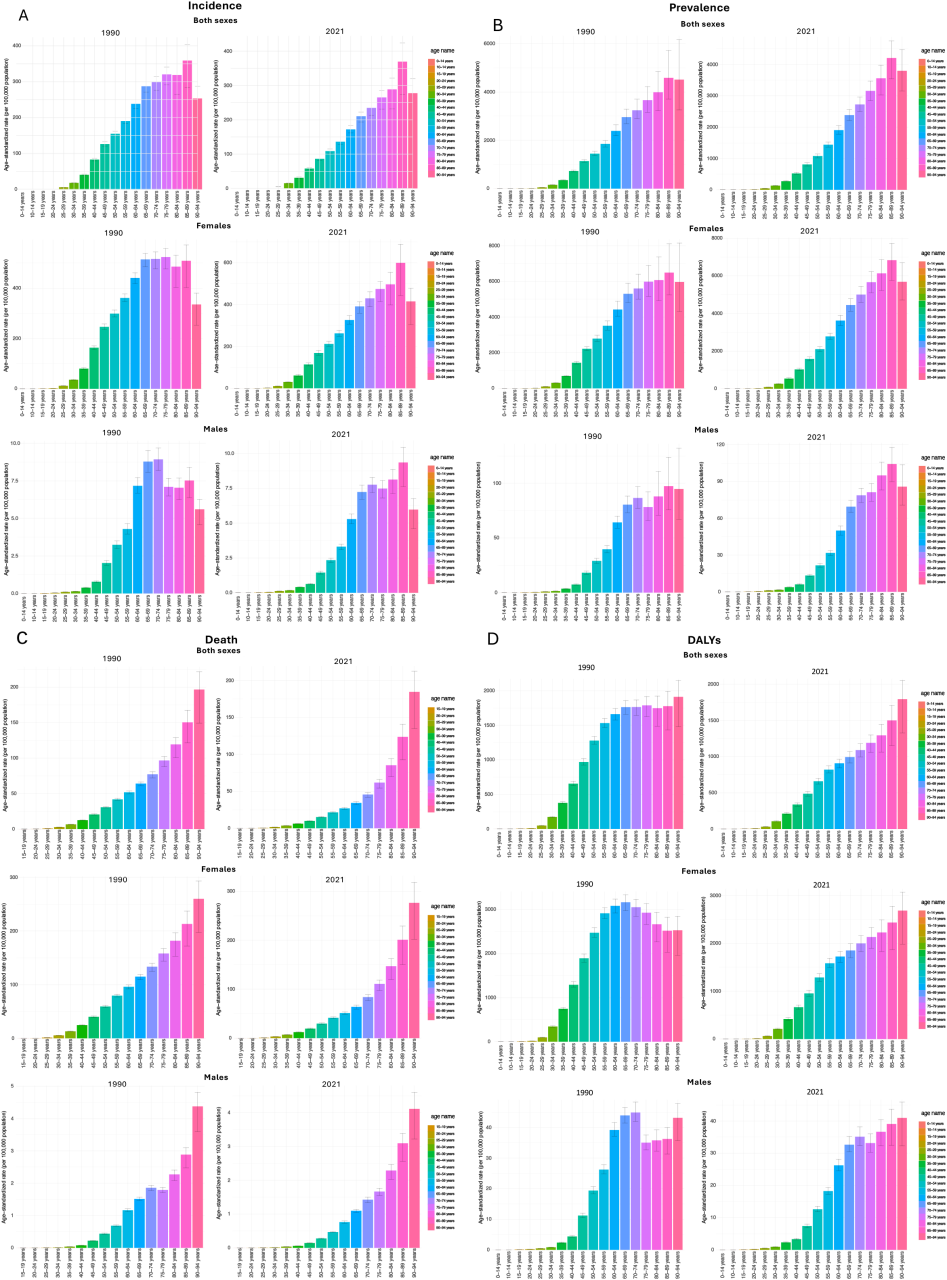
BC age-standardized incidence **(A)**, prevalence **(B)**, death **(C)**, and DALYs **(D)** rates (per 100,000 population) in 1990 and 2021 based on age groups in the U.S.

Age-standardized rates (per 100,000) for prevalence, incidence, deaths, and DALYs generally increased with age in both 1990 and 2021, with overall rates being lower in 2021 than in 1990. These trends were also observed when separately examining the changes of the rates for females and males. Specifically, the highest overall prevalence and incidence rates were estimated in the 85–89 age group in both 1990 (prevalence: 4586.2 [3705.1, 5722.4]/100,000); incidence: 359.1 (284.0, 403.6)/100,000) and 2021 (prevalence: 4205.9 [3542.8, 4754.1]/100,000; incidence: 369.6 (273.3, 423.7)/100,000) (**Supplementary Table 3**). Females were more likely to be diagnosed with BC at younger ages than males, particularly between 35 and 59 years old. For males, ASIR peaked at the age group 70-74, with 8.9 (8.2, 9.7)/100,000 in 1990 and 7.8 (7.2, 8.3)/100,000 in 2021, and then declined in older age groups. For females, rates remained at a plateau after reaching a high level starting at age 65. However, both sexes showed a noticeable drop in incidence for the 90–94 age group (**Figure 3A**).

The ASDR for both sexes, females, and males showed upward trends by age groups in both 1990 and 2021, while the death rate always reached the highest point in the 90–94 age group, and this increase was more remarkable in men while women experienced a more gradual rise. In 1990, the highest ASDR was 196.6 (149.3, 221.7)/100,000 and dropped to 184.6 (135.0, 212.5)/100,000 in 2021. For males, the measure in 1990 (4.4 [3.6, 4.8]/100,000) was also higher than in 2021 (4.1 [3.2, 4.6]/100,000). Conversely, for females, the top ASDR was higher in 2021, which was 276.4 (202.0, 318.3)/100,000, compared to the estimate of 259.9 (197.2, 293.1)/100,000 in 1990 (**Supplementary Table 3**). For DALYs, the gender differences were more distinct in the patterns and years, and the by-sex trends are also different than the overall trends for both sexes (**Figure 3D**). Data on all-age number of all burden measures in 1990 and 2021 of all age groups were listed in **Supplementary Table 4**, separated by three sex groups (both sexes, females, and males). Bar graphs of age-standardized rates (per 100,000 population) of prevalence and death of all age groups by three sex groups (both sexes, females, and males) in 1990 and 2021 can be found in **Figures 3B, C**.

### 3.4 Breast cancer burden by SDI

**Figure 4:**
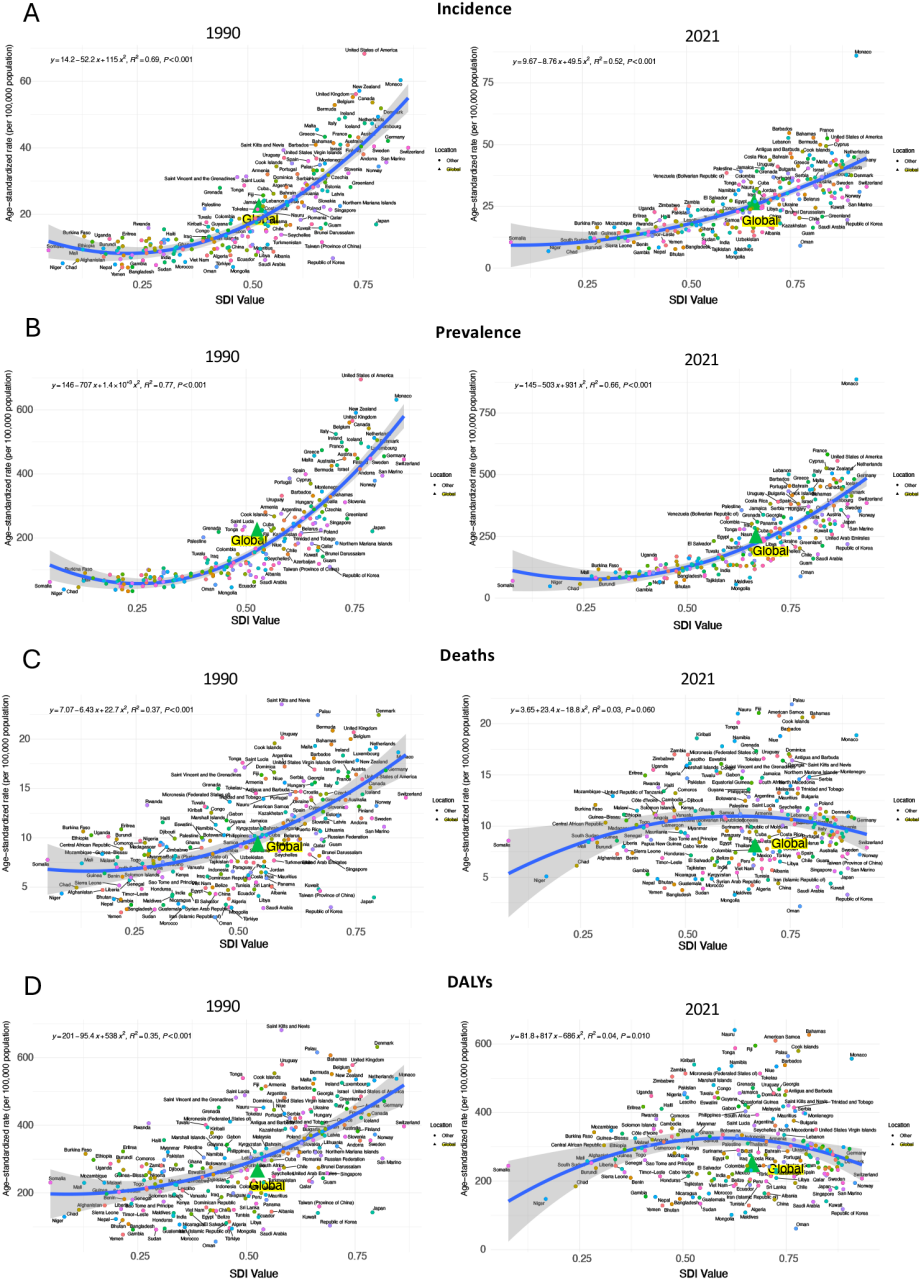
BC age-standardized incidence **(A)**, prevalence **(B)**, deaths **(C)**, and DALYs **(D)** rates (per 100,000 population) by socio-demographic index (SDI) in 1990 and 2021 worldwide.

**Figure 5:**
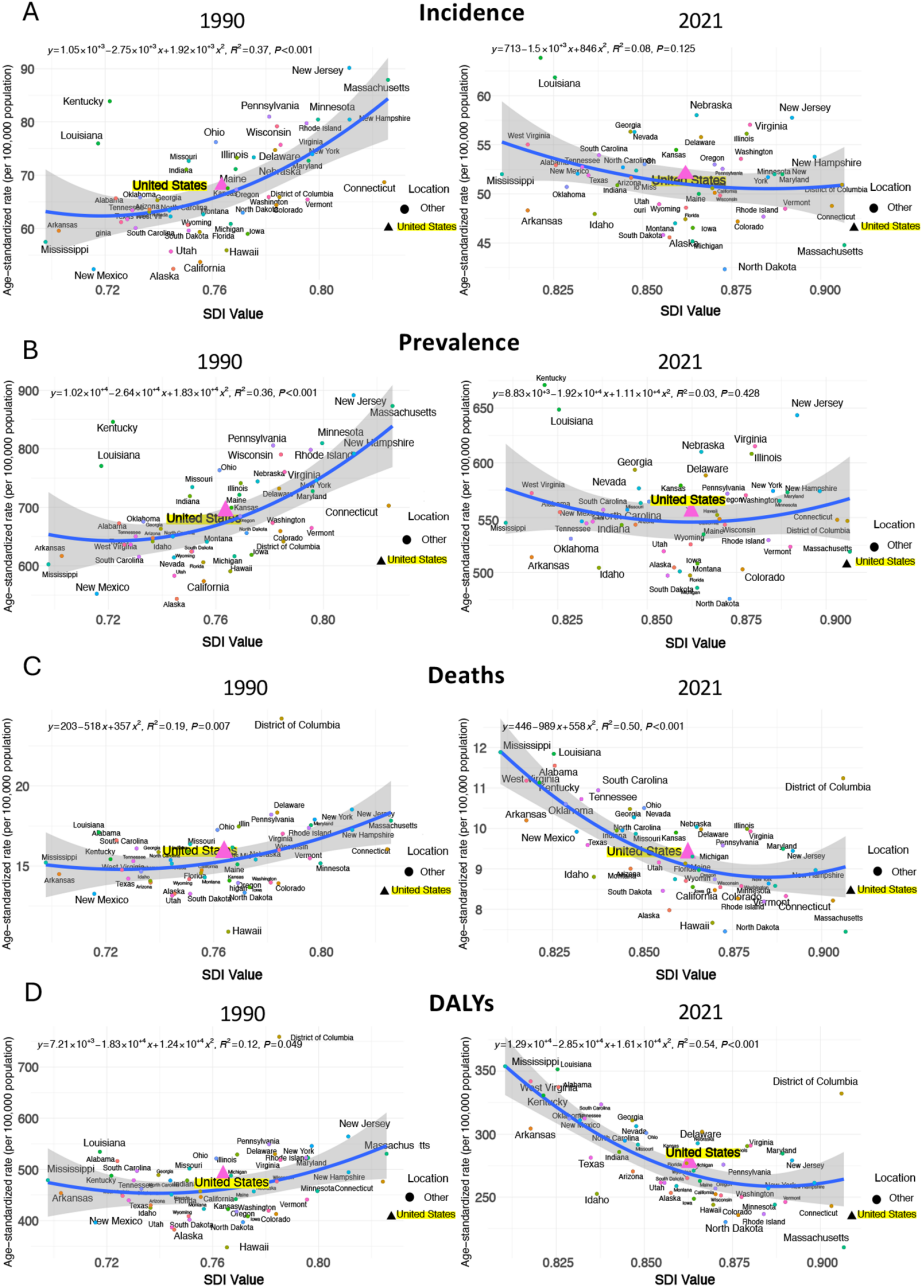
BC age-standardized incidence **(A)**, prevalence **(B)**, deaths **(C)**, and DALYs **(D)** rates (per 100,000 population) by socio-demographic index (SDI) in 1990 and 2021 in the U.S.

**Figure 6:**
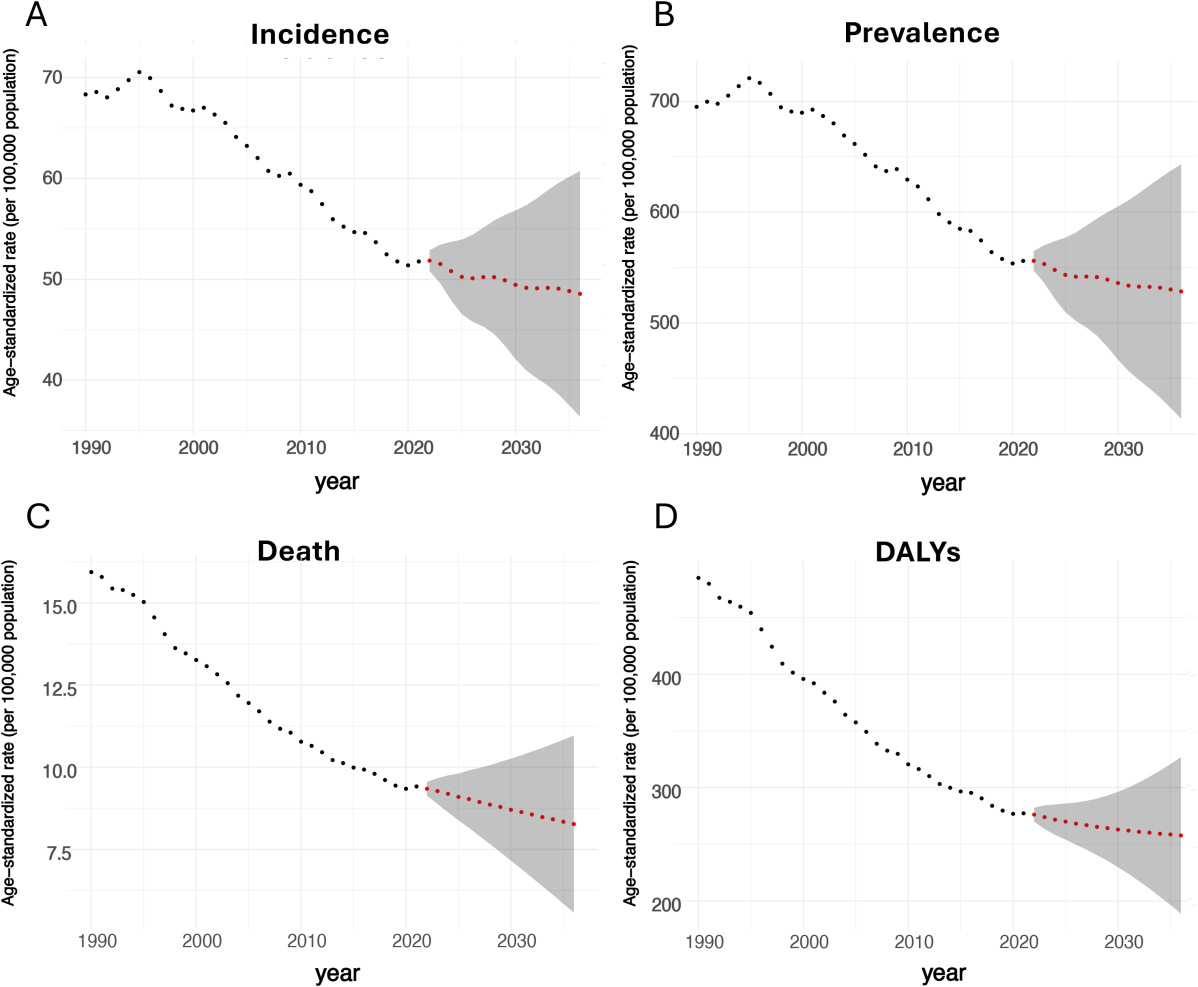
Predictions of the BC burden from 2022 to 2036 based on the ARIMA model. (**A**: predicted ASIR; **B**: predicted age-standardized prevalence rate; **C**: predicted ASDR; **D**: predicted age-standardized DALYs rate).

Globally, age-standardized rates of prevalence, incidence, death, and DALYs generally increased with SDI in 1990. However, by 2021, this pattern had slowed, with a smaller rise in burden measures at higher SDI levels, and a decline in deaths and DALYs once SDI surpassed certain thresholds (**Figure 4**). In 1990, U.S. and Monaco, as countries with relatively high SDI, had the highest prevalence and incidence rates compared to all other regions in 1990. However, by 2021, the rates in the U.S. had declined, leaving Monaco with the highest rates. For rates in deaths and DALYs, the burden of U.S. was always lower than countries with same level of SDI in both 1990 and 2021 (**Figure 5**). As a high-SDI country, the U.S. showed trends that generally aligned with global patterns, although the decline in burden over time was more pronounced in the U.S. The relationship of SDI in 1990 and 2021 with age-standardized rates of prevalence, incidence, death, and DALYs of all US states are demonstrated in **Figure 6**. All states had increased SDI and lower values for these measures in 2021 compared to 1990. In 1990, states with low to mid-level SDI, such as Alaska, Hawaii, and New Mexico, had the lowest estimates, with the exception of Kentucky and Louisiana, which had relatively low SDI but higher prevalence and incidence rates. Heavier burdens were observed in higher SDI states, like New Jersey and Massachusetts, with the District of Columbia showing notably high mortality at a mid-high SDI level. By 2021, these patterns had shifted significantly, with states having lower SDI bearing a heavier burden. Kentucky and Louisiana still had the highest prevalence and incidence rates, while Louisiana and Mississippi had the highest death and DALYs rates, all of which were states with lower SDI. In contrast, the BC burden in higher SDI states like Massachusetts had decreased compared to 1990. However, New Jersey still reported relatively high prevalence and incidence rates, and the District of Columbia continued to have notably high death and DALYs rates.

### 3.5 Forecast results for 2022-2036

The ARIMA model forecasts a gradual decline in the age-standardized rates (per 100,000) for prevalence, incidence, deaths, and DALYs for BC from 2022 to 2036 in the entire U.S. for population of both genders (**Figure 6**). ASIR was expected to decrease to 48.6/100,000 by 2036, representing a 6.0% drop from the 2021 rate of 51.7 (48.4 - 54.1)/100,000. Regarding prevalence, the estimated level in 2036 will be 528.5/100,000. For ASDR, it was projected to keep dropping to 8.3/100,000 in 2036, indicating a 11.7% drop from the 9.4 (8.5 - 9.9)/100,000 in 2021. In terms of DALYs rate, it is forecasted to decrease to 257.8/100,000 in 2036.

## 4 Discussion

This study assessed the burden of BC and its trends in the U.S. from 1990 to 2021 using the GBD 2021 database. During the study period, age-standardized prevalence, incidence, mortality and DALY rates for breast cancer declined; nevertheless, the absolute numbers of cumulative cases and deaths in the United States rose. This apparent paradox was driven predominantly by demographic expansion and population ageing. The increase in case counts mirrored growth in the total US population and a shift in the age structure toward older adults— the stratum in which breast cancer most frequently occurs. The downward trend in age-standardized rates indicates that, after removing the influence of these structural changes, the true epidemiological burden of breast cancer in the United States has in fact diminished. Unlike the global trend of increasing burden on BC, the U.S. has shown progress on the prevention and treatment for this common cancer. However, the total numbers for these measures have been increasing over time, except for DALYs, which showed a recent upward trend following a decline in previous years. The reduction in BC incidence and mortality has been reported in the 2020 GLOBOCAN online database (26) and the 2022 BC Statistics from the American Cancer Society’s updates (27), as well as in other studies (6, 28), though these sources only addressed BC mortality in women. While previous studies focused predominantly on female BC, our analysis comprehensively includes males when evaluating trends of BC burden in the U.S. Although trends by sex were generally aligned with national patterns, females consistently experienced a significantly higher BC burden than males from 1990 to 2021, which is concordant with the widely revealed finding about the comparison of BC burden for both sexes, that men count for about 1% of BC cases (7, 29).

High prevalence and incidence burdens were notably observed in specific states, with New Jersey in 1990 and Kentucky in 2021 consistently showing high rates for both sexes. Conversely, lower burden measures were recorded in various states in both years, with Hawaii and North Dakota being reported more frequently than states like Alaska, Michigan, and Massachusetts. Despite some state-level variations over time, the overall trend indicated a decline in BC prevalence and incidence across the U.S., suggesting that preventive measures and treatment approaches have been effective. Even though access to mammography and BC screening programs can potentially lead to earlier and higher rates of disease detection in certain states, the disparities in incidence can also be influenced by several risk factors, such as smoking (30, 31), high alcohol consumption (32, 33), obesity (28), and limited physical activity (34). Furthermore, variations in population demographics, including age, race, and ethnicity (4), play a role. To address these disparities, states with high BC incidence should prioritize comprehensive health management and targeted education, focusing on improvement of screening accessibility and participation, and addressing modifiable risk factors through public health initiatives. Additionally, the patterns differed between females and males, with the highest and lowest age-standardized prevalence rates and ASIR reported in different states for each sex, highlighting potential regional health disparities between genders.

The mortality burden is notably more pronounced in specific states. In 1990, the highest ASDR and age-standardized DALYs rates were observed in the District of Columbia. By 2021, the highest rates had shifted to Mississippi. Conversely, the lowest rates were consistently recorded in Hawaii in 1990 and in Massachusetts in 2021. Additionally, states such as Louisiana and Hawaii were also noteworthy in the data. The findings match the observation for certain states, for example, Hawaii has maintained a low mortality rate in BC for decades (35). The observed variations in mortality rates among U.S. states can be significantly influenced by differences in healthcare access and quality. States with more robust healthcare systems and better access to medical services, including early detection and treatment, typically report lower mortality rates. The disparities in healthcare infrastructure and insurance coverage across states can greatly impact health outcomes, leading to varying levels of mortality burden.

Socioeconomic factors play a crucial role in shaping health outcomes. If taking into SDI into account when examining the BC burden by states, we observed similar trends on the relationship between age-standardized rates for prevalence, incidence, death, and DALYs with SDI. Consistent with the state-level heterogeneity observed in the United States, the Global Burden of Disease (GBD) study also documented marked between-region heterogeneity in the population attributable fractions (PAFs) of breast-cancer risk factors across Socio-demographic Index (SDI) quintiles. For example, high body-mass index and alcohol use generally exhibited higher PAFs in high-SDI countries. These findings indicate that, despite differences in underlying incidence rates, health inequalities driven by socioeconomic disparities within the United States are comparable to the global pattern. States at both high and low ends of the SDI spectrum tended to have higher incidence and mortality burdens, while states with mid-level SDI consistently showed the lowest estimates. Even so, burden measures were decreasing in high-SDI states. The trends found in the U.S. aligns with the global pattern where BC mortality burden, as indicated by ASDR and DALYs, has decreased worldwide over time, particularly in high and high-middle SDI regions. From 2010 to 2017, a phenomenon was reported that those regions with low-middle SDI exhibited higher burden levels compared to those with high SDI (32). Advances in treatment, including cutting-edge surgical technologies, radiation therapies, and targeted systemic agents tailored to BC subtypes in developed countries, could potentially explain the reduction in ASDR and DALYs rates (36). Furthermore, regions with low SDI are predicted face the highest BC burden in the future (16), which is often linked to economic development that affects access to healthcare resources and infrastructure. In contrast, high-SDI states, which face fewer of these challenges, are more likely to experience a high burden due to lifestyle-related, environmental, and occupational risk factors. For example, high level of alcohol consumption was found in high-SDI regions globally (37). Given these findings, U.S. policymakers should consider targeted strategic actions to address the anticipated disparities in BC burden in different regions. Enhancing healthcare access and infrastructure in economically disadvantaged areas can be the priority, including increasing the availability of screening programs, diagnostic services, and treatment facilities to improve early detection and management of BC. For states with higher SDI, effort should be put on reducing lifestyle-related risk factors, promoting healthier diets, and encouraging regular physical activity. Additionally, policies aimed at reducing exposure to environmental and occupational toxins should be strengthened, particularly in industries and regions with higher exposure risks.

In terms of age, we found that the age-standardized rates of prevalence, incidence, deaths, and DALYs associated with BC exhibited an overall increasing trend across certain age groups in both 1990 and 2021. This age-specific pattern is broadly consistent with the global GBD estimates for breast cancer; however, the increase in age-specific incidence among younger women was more attenuated in the U.S. The highest prevalence rates were frequently observed in the 85–89 age group for both genders, but for incidence rates, it was always the 70–79 age groups who had the highest risk of developing new BC cases for men and women. Younger populations, particularly men under 69, experienced the sharpest rise in incidence rates compared to older age groups. This finding somehow aligned with the previous studies at the global level, revealing that the increase in ASIR diminishes with age, with females under 50 experiencing the greatest rise (38). In contrast to incidence trends, older populations consistently bore a higher mortality burden. The 90–94 age group, in particular, experienced a significantly greater ASDR compared to younger age groups. However, trends in DALYs by age group did not show such a direct correlation, with the highest rates observed in the 65–74 age group while older age groups even had lower or unchanged DALYs rate levels. Therefore, the practical implication of these findings is that more preventive efforts for BC can be put in for younger age groups, which aims to reduce incidence rates and, consequently, lower mortality burden as they age, leading to better overall health outcomes and less burden for the entire society. Key strategies should include raising general awareness, implementing and maintaining effective screening programs, and addressing preventable risk factors such as lifestyle and behavioral changes, stress, and socioeconomic conditions.

Although substantial domestic heterogeneity in breast-cancer burden persists and the absolute number of cases keeps rising because of population growth and ageing, all summary indicators are projected to continue their favorable downward trajectory. Compared with other major female malignancies in the United States, the decline in breast-cancer burden has been particularly pronounced. This observation reflects a genuine epidemiological risk reduction attributable to advances in both prevention and treatment, and it underscores the need to intensify efforts to further alleviate the breast-cancer burden among the U.S. population.

Our study has some limitations. GBD study provides high-quality burden estimates for various diseases, there are still some challenges that might impact the unbiasedness and accuracy of the findings represented using the GBD database. The study largely depends on data from vital and cancer registries, as well as other epidemiological studies worldwide. However, data collection infrastructures are not uniformly developed across the world, or even within the U.S., particularly in economically disadvantaged regions (39). This uneven development can affect the quality of data and the reliability of the analysis in these areas (40). The U.S. holds an advantage in that it has a longer history of developing cancer registries and implementing public health programs compared to many other countries. This has led to a more advanced and effective use of cancer registry data (41).

This study provides BC burden estimates and makes comparisons based on common factors available in the GBD data, such as gender, age, geographic regions, and SDI. However, it does not investigate deeper into the specific contributing factors, such as alcohol consumption, tobacco use, physical activity, and diet, or their impact on each measure of BC burden (42). Future studies could assess the contribution of various attributable factors to BC burden estimates, both overall and within different demographic groups. This would provide valuable insights for more effective risk factor management in BC prevention. Furthermore, the natural processes of population growth and aging can impact burden estimates to some extent, and our study did not analyze these factors separately. Future research could use techniques such as decomposition analysis to gain a more accurate understanding of BC burden, which would support more informed and precise policymaking.

## 5 Conclusion

In conclusion, the burden of breast cancer in the U.S. has decreased significantly between 1990 and 2021, largely due to advancements in detection, treatment, and public health initiatives. And projections for the next 15 years suggest a continued decline. However, disparities persist across population groups of different gender and age groups and states, particularly in lower SDI regions. Targeted interventions and improved healthcare access remain essential to addressing these inequalities. Continued efforts are needed to reduce the burden and promote health equity nationwide.

## Data Availability

The original contributions presented in the study are included in the article/**Supplementary Material**. Further inquiries can be directed to the corresponding authors.
