# Supplementary figures and images for: Breast cancer burden in the United States (1990–2021) with a 15-year forecast: a comprehensive analysis based on the global burden of disease 2021
Source: Front Oncol. 2025 Nov 13;15:1650110. doi: 10.3389/fonc.2025.1650110 (PMC12657151; doi:10.3389/fonc.2025.1650110)

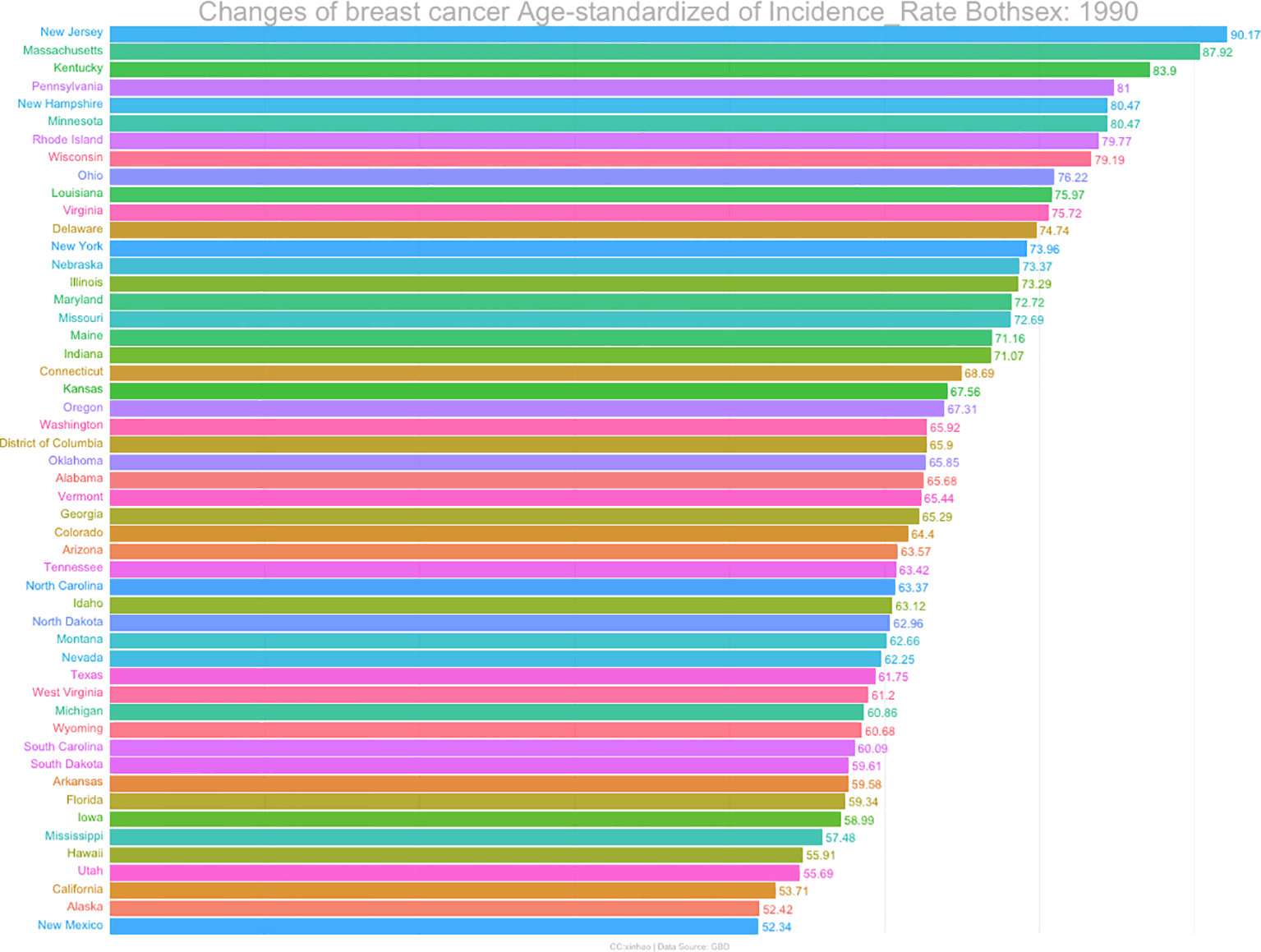

Supplement: Supplementary file 5 [file Supplementaryfile1.gif]

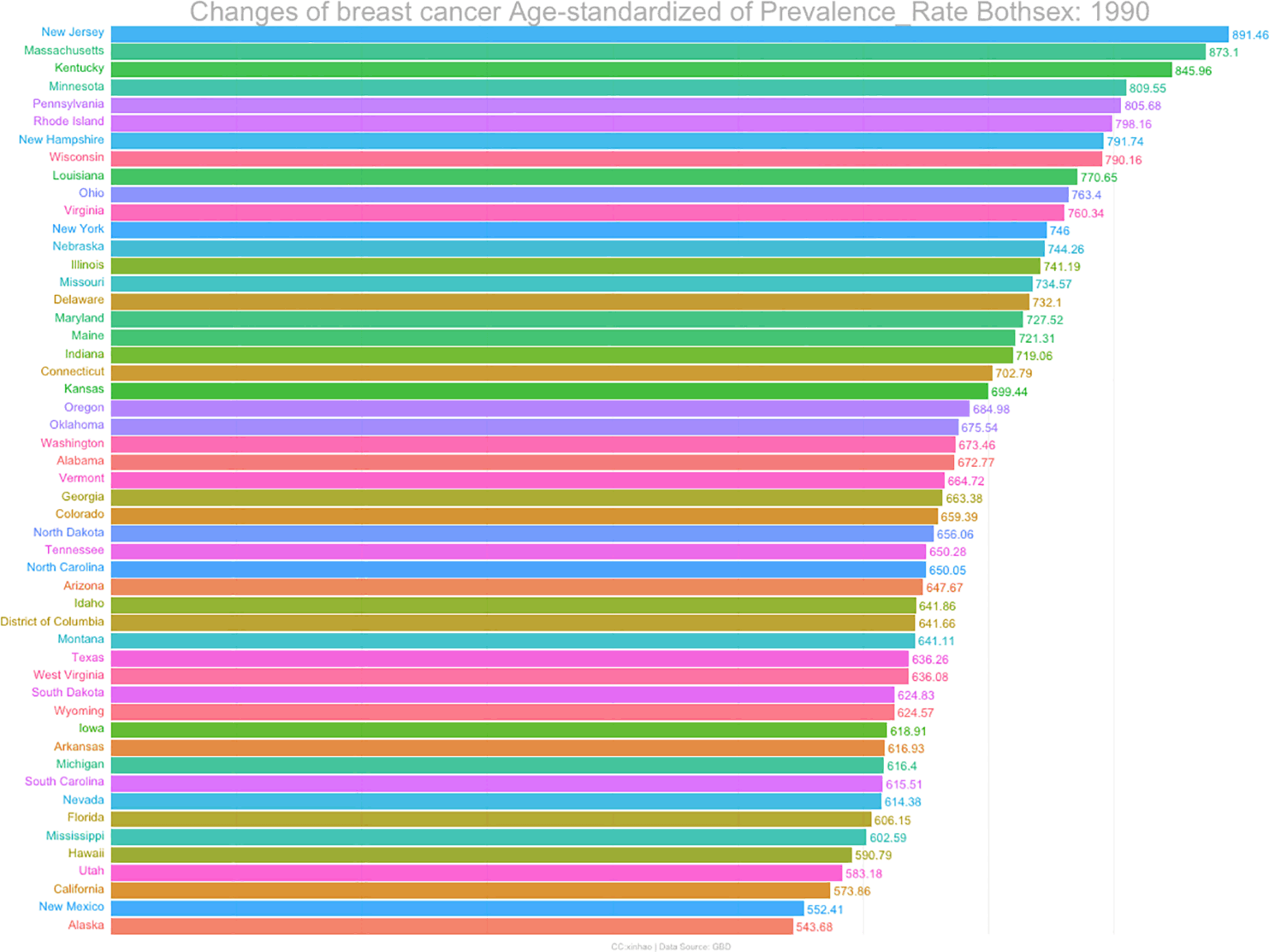

Supplement: Supplementary file 6 [file Supplementaryfile2.gif]

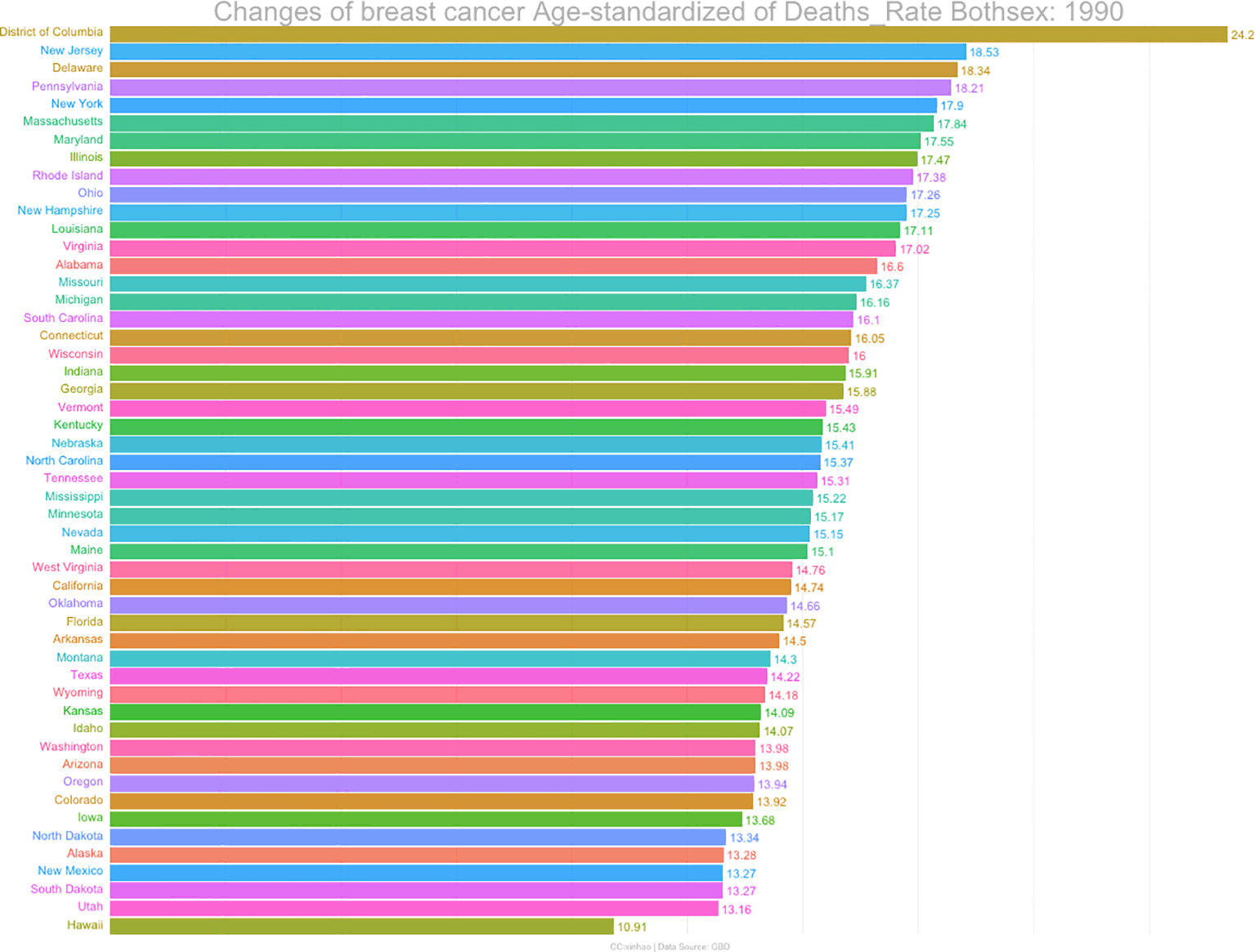

Supplement: Supplementary file 7 [file Supplementaryfile3.gif]

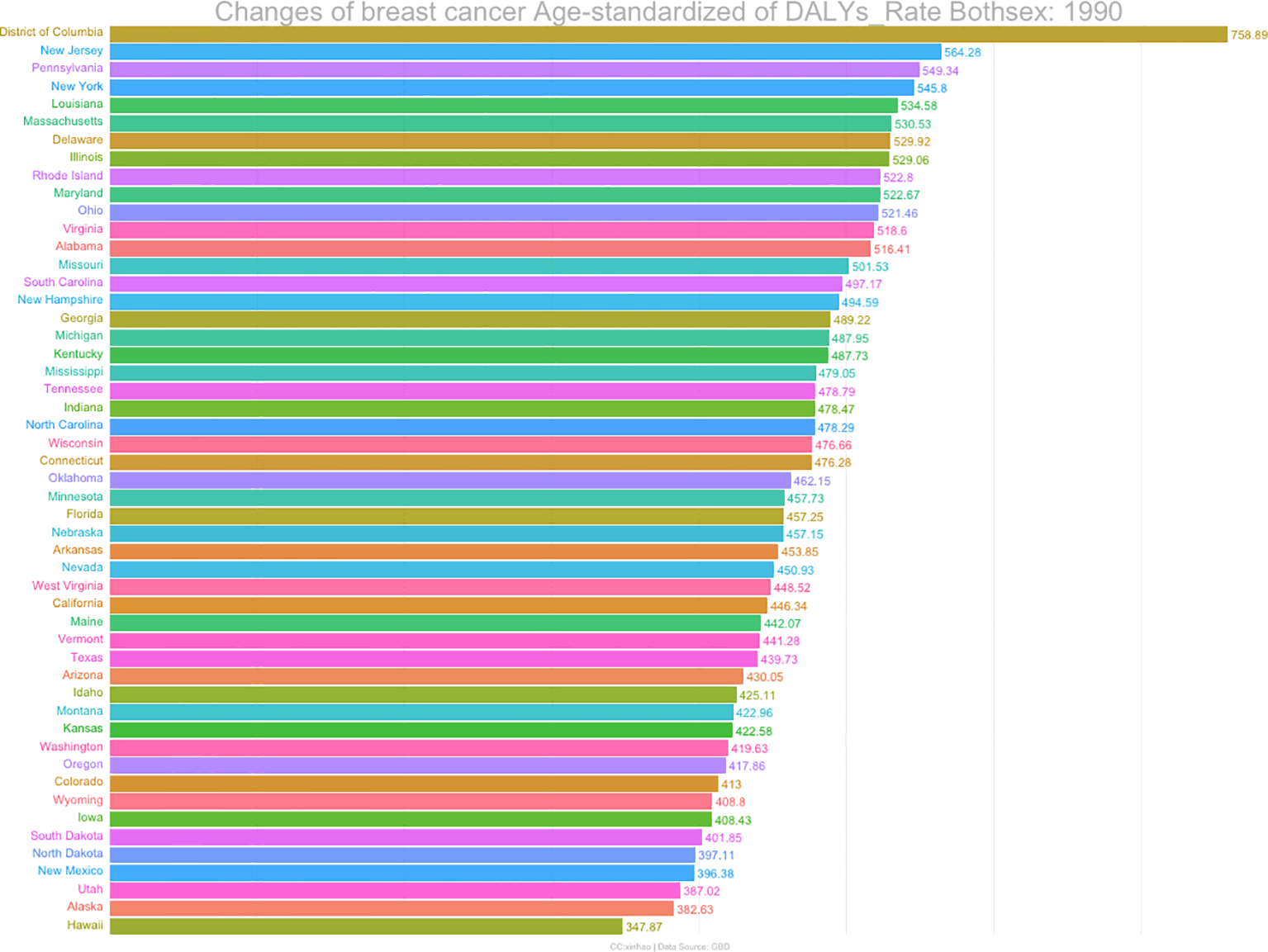

Supplement: Supplementary file 8 [file Supplementaryfile4.gif]
